# Rapid Quantitative Fluorescence Detection of Copper Ions with Disposable Microcapsule Arrays Utilizing Functional Nucleic Acid Strategy

**DOI:** 10.1038/s41598-018-36842-x

**Published:** 2019-01-10

**Authors:** Enqi He, Liangyuan Cai, Fengyi Zheng, Qianyu Zhou, Dan Guo, Yinglin Zhou, Xinxiang Zhang, Zhihong Li

**Affiliations:** 1 State Key Laboratory of Tribology, Department of Mechanical Engineering, Tsinghua University, Beijing, 100084 China; 2 Beijing National Laboratory for Molecular Sciences (BNLMS), Key Laboratory of Bioorganic Chemistry and Molecular Engineering of Ministry of Education, College of Chemistry, Peking University, Beijing, 100871 China; 3 National Key Laboratory of Science and Technology on Micro/Nano Fabrication, Institute of Microelectronics, Peking University, Beijing, 100871 China; 4 Center for Nano and Micro Mechanics, Tsinghua University, Beijing, 100084 China

## Abstract

In this work, an economical and easy-to-use microcapsule array fabricated by ice printing technique has been realized for ultrasensitive fluorescence quantification of copper ions employing functional nucleic acid strategy. With ice printing, the detection reagents are sealed by polystyrene (PS) film isolation and photopolymer, which guarantees a stable and contamination-free environment for functional nucleic acid reaction. Our microcapsule arrays have shown long-term stability (20 days) under −20 °C storage in frozen form before use. During the Cu^2+^ on-site detection, 1 μL sample is simply injected into the thawy microcapsule by a microliter syringe under room temperature, and after 20 minutes the fluorescence result can be obtained by an LED transilluminator. This method can realize the detection limit to 100 nM (100 fmol/μL) with high specificity.

## Introduction

DNA is normally regarded as a carrier of genetic information, but, in fact, it can serve far more functions. The synthesis from DNA complex into nanostructures can be automatically formed through programmed combination based on the basic Watson-Crick complementarity of A-T and C-G base pairing^1,2^. As a sensing strategy, DNA aptamers are widely used due to the effectiveness of binding any molecule of choice^3,4^. DNAzyme is regarded as another kind of functional DNAs, since it will catalyze the cleavage of substrate DNA when certain metal ions present^5,6^. In virtue of this characteristic, when the metal recognition substrate is linked with a signal generation moiety such as a fluorophore, the designed DNAzymes can be applied as sensors of specific metal ions^7–10^. This strategy attracts great attention because of its ultrasensitive property of metal ion quantification. Although there are several methods, such as atomic absorption spectroscopy, inductively coupled plasma atomic emission spectrometry, electrochemical sensoring, and the use of piezoelectric quartz crystals, making it possible to detect low limits^11–14^, expensive equipment, time-consuming procedures and well-trained analysts are necessary. On the contrary, DNAzyme can realize real-time and on-site detection of metal ions revealing great popularity. However, essential conservation of detection system avoiding inactivation and contamination needs to be taken into account through actual application process.

Commonly found in nature, the copper ion can be intaken by human body in the form of drinking water. Maintaining the copper ion at a relatively low level is important for keeping human body function. However, a high concentration may cause diseases. Exposure to high level of copper ion for a short period of time will lead to gastrointestinal disturbance, and the functions of human liver and kidney can be detrimentally influenced with constant ingestion^15^. According to National Primary Drinking Water Regulations of USA, 1 mg/L (1.5 pmol/μL, ~20 μM) is the maximum of copper ion in drinking water. Therefore, low-cost on-site chips which can offer people quantitative real-time detection of copper ions with identifiable signals are clearly imminent.

Chips that are potential to various analytical assays without the help of sophisticated equipment and laboratorial conditions are known as lab-on-a-chip technology^16,17^. Many devices have been developed in recent years, among which microfluidics is the main focus of research. Normal microfluidic chips are fabricated on silicon wafers by two or three dimensional MEMS process, including photolithography, laser photoablation, and chemical etching^18–20^. In spite of delicate devices with precise micro-scale channels and high level of integration, micromachining difficulties and optical opacity limit their applications. In addition, toxic residues from microfabrication processes make it hardly suitable for biological and biomedical uses. To meet this need, glass and polymer based microfluidic chips that are transparent and biocompatible are developed for various bio-applications. The fabrication of these chips, generally known as soft lithography, pouring liquid precursor of polymers such as polydimethylsiloxane (PDMS) onto a machine-fabricated channel mold or sacrificial layer, curing the polymer into solid and binding onto glass surface, is simply accessible^21,22^. Nevertheless, since the price of material polymer is still high and the fabrication efficiency is limited, it is difficult to put microfluidic chips into mass production as disposable devices for daily use.

Paper-device, one typical disposable device, has been proved a great success in commercial market. As a superior approach, it features high reaction rate, low sample consumption and great biocompatibility^23^. Although breakthroughs have been made in paper-based bioanalysis, including immunochromatographic testing paper devices and paper-based microfluidic bioassays^24–27^, further complicated bioassays with fragile reagents, especially those concerning DNA-related reaction, are restricted to combine with paper-based devices. Firstly, fragile reagents need to be isolated from open air in a sealing environment, because deactivation of reagent system may occur if contamination or oxidation happens. Secondly, liquid-phase incubation is necessary for some detection strategies, including DNAzyme technique. In addition, sensitive signals of assays, for example fluorescence widely applied in detection methods^28–33^, are more likely to be observed in liquid. Thirdly, we always expect target determinations the more sensitive the better, however, paper-based analysis hardly conducts accurate quantitative results with low detection limits, for the reasons including two limitations discussed above and capillary action of sample introduction.

In some previous work, methods of copper ion detection based on microfluidic and paper-based devices have been published^34–37^. For microfluidic devices, although the detection results are convincible, their operations are complicated and the cost for disposable devices is high, which means on-site daily application is hard to get achieved. Regarding paper-based devices, it is convenient for daily uses due to the low cost of device and easy operations, however, detection sensitivity and stability need to be improved. With the technique of 3D ice printing, our group initially invented a disposable, low-cost and anti-contaminating microcapsule array chip^38^. A small volume of reaction solution is printed onto a cold substrate and frozen into ice droplet, which acts a mold for the next step of photopolymer pouring. Then, light-cure photopolymer is poured covering the ice and solidifies under LED to seal the reaction solution. After fabrication, the microcapsule chip is transferred to −20 °C immediately for storage. In actual uses, a small amount of sample is injected into melted microcapsules and detection result can be read out through colorimetric method. This novel chip is advantageous in bioanalysis for daily uses due to following traits. Firstly, unlike other reaction solution conserved devices in which liquid system is infused after fabrication, the reaction solution is pre-sealed in ice printed chips during fabrication preventing the reagents from secondary contamination. Secondly, the whole fabrication process is biocompatible and pollution free, which won’t do harm to the reagent biological activity. Indeed, the freezing treatment and storage actually protect the reaction solution from deactivation. Thirdly, the “bottom-up” fabrication of printing makes it potential to mass production ensuring its disposable and cost-effective properties.

In this work, we first developed disposable microcapsule arrays based on ice printing fabrication and functional nucleic acid sensing strategy for real-time on-site fluorescence quantification of copper ions with ultrahigh sensitivity. However, the previously developed microcapsule array chip is not suitable in this assay, because when the microcapsules melt for application the residual of photo-polymerization may intrude into liquid-phase reaction solution from solidified photopolymer damaging the bioactive DNA-based reagents, and thus interfere the quantification sensitivity and accuracy. To deal with this issue, we improve the ice printing fabrication process with an addition of polystyrene (PS) film forming procedure, which isolates the fragile reagents from photopolymer. The PS film is formed by a printing process as well, particularly including two steps: Firstly, PS solution is dropped to fully cover the iced reaction solution. Secondly, the solvent of PS solution completely volatilizes, forming a dense PS film. In our experiment, the PS film isolating the reaction solution and photopolymer efficiently protects DNAzyme system contributing to the realization of on-chip ultrasensitive fluorescence quantification of copper ions.

## Results

### Fabrication of microcapsule arrays and Theory of copper ion determination

**Figure 1:**
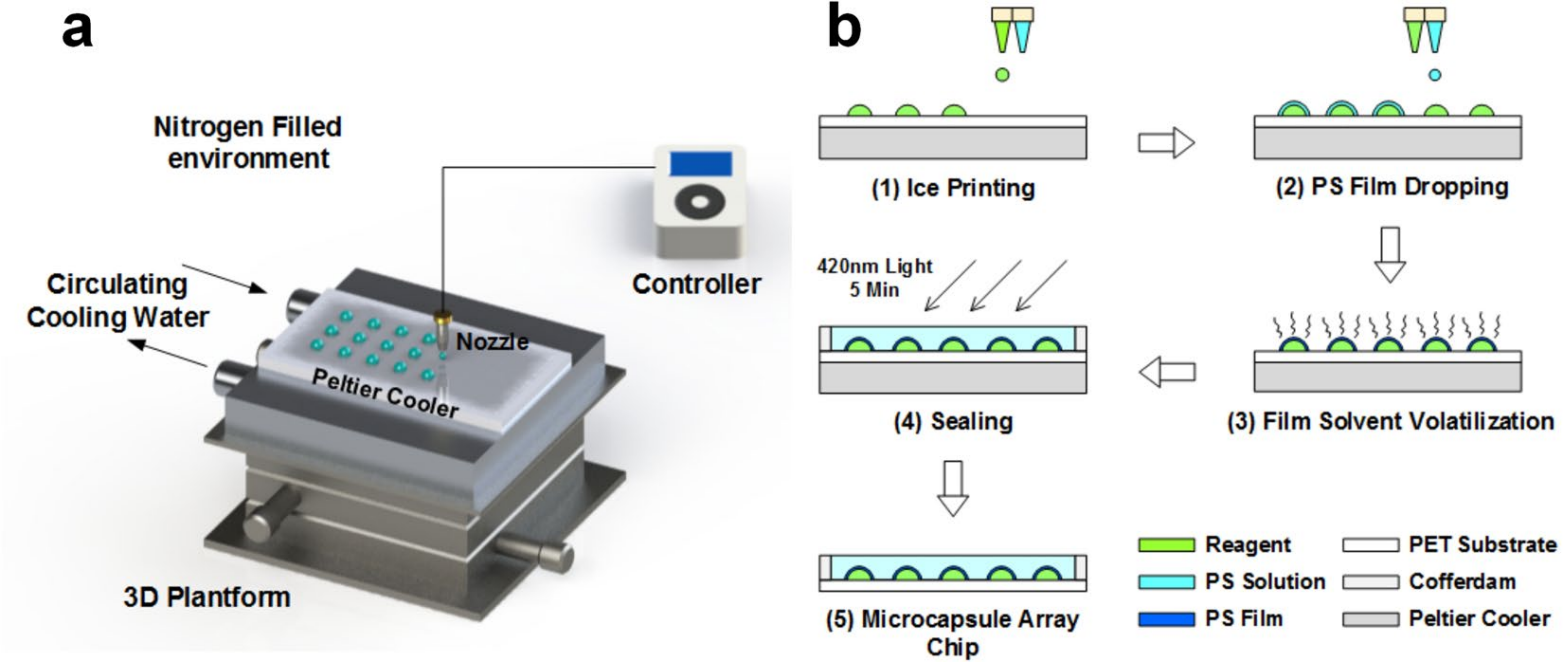
Fabrication of Microcapsule arrays. (**a**) The fabrication system of ice printing. (**b**) Step-by-step procedure of fabricating microcapsule arrays in this work.

The fabrication of microcapsule arrays, including the ice-printing system (a) and the fabrication procedure (b), is demonstrated in Figure 1. Under the command of controller, the printing nozzle ejects reaction solution. The location of droplets on the substrate is determined on 3D platform operated by human hands. The Peltier cooler can cool the substrate to below −20 °C with heat dissipation of circulating cooling water. The detailed fabrication procedure can be concluded as five steps. (1) 10 μL reaction solution composing of Cu-substrate, Cu-enzyme, ascorbate and corresponding buffer is printed onto a certain spot of the polyethylene terephthalate (PET) substrate determined by 3D platform. When the controller sends the printing command, the nozzle starts to print the reaction solution from about 2 mm above the PET substrate and moves upwards slowly to ensure the reaction solution continuously falling onto the droplet surface. This procedure lasting 3 seconds guarantees no reaction solution remaining on the nozzle and a uniform shape of each droplet unaffected by its falling-down gravity. After 10 μL reaction solution is all printed on the PET substrate, it is then frozen into ice under −20 °C caused by the Peltier cooler. These ice droplets act not only as the reaction system of Cu^2+^ fluorescence detection but also as the solid template for microcapsule fabrication. Besides, the solid state of ice droplets guarantees that the following fabrication procedures would not harm to the fragile reaction system, since no reactions, neither physical nor chemical, would occur on the ice surface. (2) 8 μL previously prepared PS solution (27.5% PS in chloroform) is dropped by another nozzle (2 mm above the top of the ice droplet surface). The dripped PS solution is thick enough to encapsulate the surface of ice droplets. (3) After the solvent of chloroform completely volatilizes in 15 minutes, the solute polystyrene is left forming a thin dense film wrapping up the ice droplet. Solvent volatilization conducts under −8 °C, which would shorten the time consuming of the volatilization process and maintain the ice droplets. (4) Light-cure photopolymer is added to cover the arrays and self-levelled assisted by cofferdam. Polymerization starts under 420 nm LED and the microcapsules are effectively sealed after 5 minutes. During this sealing procedure, the substrate temperature is set to −20 °C again, since the polymerization would release heat. This further sealing procedure is still necessary though the PS film encapsulates the ice droplet, since the formed PS film is very thin (about 50 μm) and not strong enough to support the vessel, especially when the reaction solution melts into liquid. (5) The obtained chips are stored in −20 °C condition, such as refrigerator, to let the microcapsules stay frozen before use. The whole fabrication process takes less than 30 minutes, which is more timesaving than most fabrication methods.

**Figure 2:**
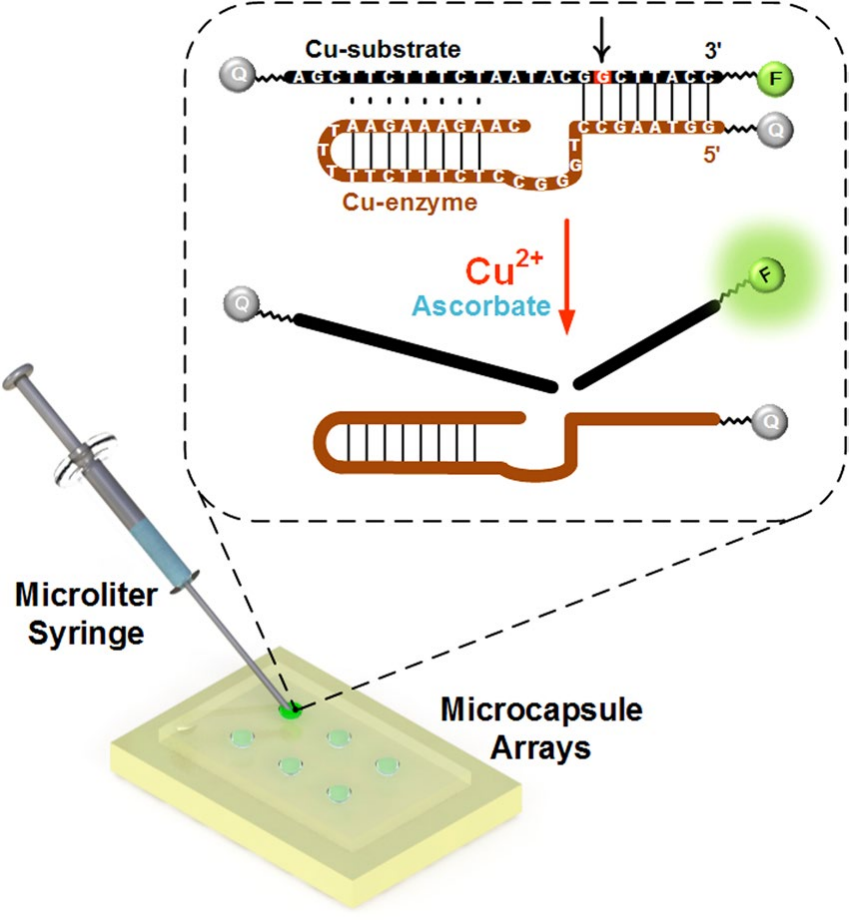
Main principle of Cu^2+^ fluorescence on-chip detection based on DNAzyme.

When in use, the chip is taken out from frozen storage environment, and iced reaction solution in microcapsules melts under ambient temperature within 1 minute. Dry the chip surface by wiping the dew up using paper tissue. By a microliter syringe, the PET substrate is punctured and 1 μL target sample is injected simply into each thawy microcapsule. The punctured surface is cleaned up by paper tissue since a little reaction solution would leak out and transparent tape is used to seal the punctured hole. In the presence of copper ions, DNAzyme cleavage reaction incubates in microcapsules on chip (Figure 2). Originally, Cu-substrate and Cu-enzyme, two rationally designed DNA strands, bind together via Watson-Crick base pairing and form a complex. The Cu-substrate has the sequence of 5′-AGCTTCTTTCTAATACGGCTTACC-3′, and is labeled with a FAM fluorophore (6-carboxyfluorescein) at the 3′ end and a quencher (BHQ-2) at the 5′ end. The Cu-enzyme has the sequence of 5′-GGTAAGCCTGGGCCTCTTTCTTTTTAAGAAAGAAC-3′, and is labeled a quencher (Dabcyl) at the 5′ end. Due to the quenching effect induced by the substrate 3′-quencher and the enzyme 5′-quencher, the FAM emission of the substrate 5′-fluorophore is suppressed. Such a dual-quencher approach is able to restrain background signals efficiently^39^. After injection, irreversibly cleavage to the Cu-substrate would get induced at the cleavage site (red point in the figure, the first guanine from the 3′ end on Cu-substrate) due to the presence of Cu^2+^ in the sample which releases the FAM fluorophore from two quenchers and leads to the FAM emission. Ascorbate, which originally exists in the reaction system, is necessary to the DNAzyme detection method because it can enhance the reaction rate and suppress quenching^40–42^. Since the FAM emission signal caused by the released fluorophores increases fast at early stage and afterwards varies little in the main (Figure S1), it can be detected under commercial portable LED transilluminator after 20 minutes. Photos are taken by cell phone and further analyzed based on the software ImageJ (Figure S2). The fluorescence value, equal to the difference between the fluorescence intensities of experimental spot and negative control, indicates fluorescence intensity enhanced by the copper ion injection. Relied on this processed fluorescence value result, copper ions in the sample can be quantified.

### PS film function

**Figure 3:**
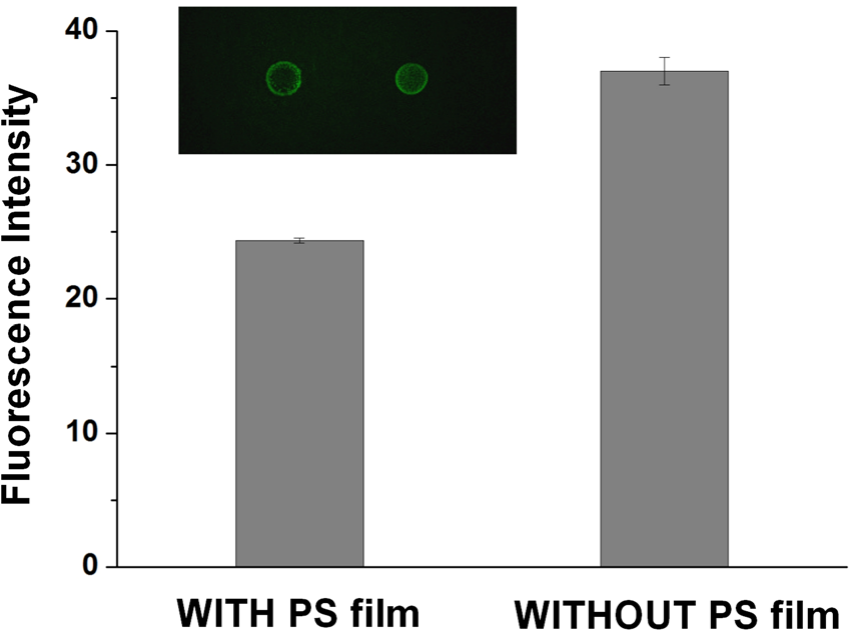
Fluorescence intensity of negative control with/without the PS film protection. In the inserted photo, the left microcapsule is fabricated with PS film, however, the right one isn’t. The fluorescence intensity of the microcapsule without PS film is higher than that of the microcapsule with PS film, indicating that the PS film protects the reaction solution inside the microcapsule.

Since polymerization residues from solidified sealing photopolymer may contaminate the thawy reaction solution losing efficiency and increase the background interference during chip usage, it is important to establish an effective protection of reaction solution against photopolymer. In this work, PS film acts as a protection film encapsulating the ice droplet before the photopolymer sealing process. During the PS film forming step, 8 μL PS solution is dripped onto the ice droplet of reaction solution, whose surface is completely covered. After the solution volatilizes, a dense coating of PS film is formed, encapsulating the droplet. Figure 3 illustrates that the microcapsule with PS film stays with low background, indicating the DNAzyme system is efficiently protected. However, without PS film, the reaction solution would emit fluorescence in the absence of copper ions, which affects the quantification assay.

### Performance of copper ion Determination

In order to conduct fluorescence quantification with high sensitivity, background fluorescence emitted by chip material shouldn’t be too strong to interfere observation result. As PS and PET are widely used as materials of ELISA plate and centrifuge tube, they show little fluorescence effect. We test the fluorescence emission spectrometry of solidified light-cure photopolymer, and it shows that there would be no fluorescence interference to the assay brought by the sealing photopolymer due to the very little emission of green fluorescence (wavelength 530 nm ~ 570 nm) under 480 nm excitation (Figure S3).

**Figure 4:**
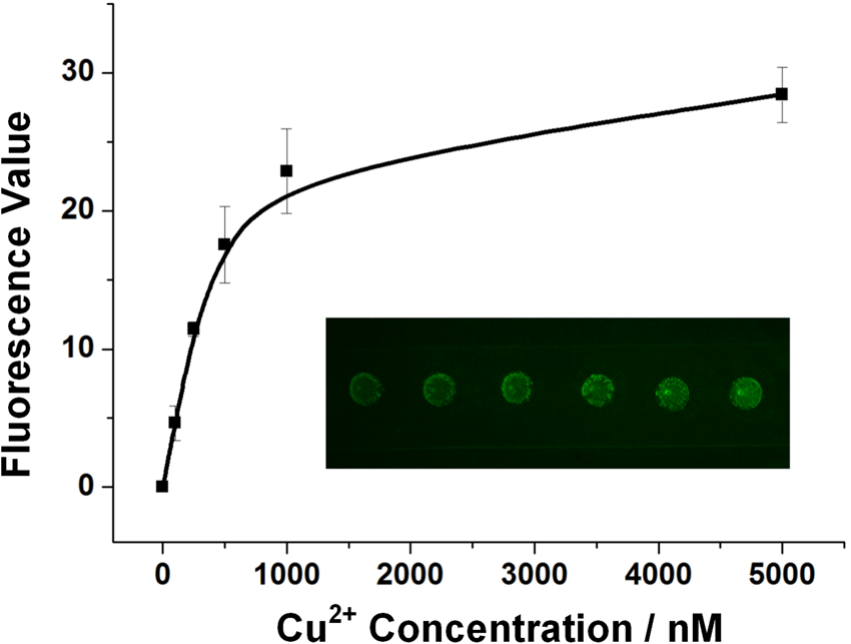
Calibration curve between fluorescence value and Cu^2+^ concentration. The inserted photo shows that six microcapsules on one chip are separately injected six Cu^2+^ solutions whose concentration range from 0 nM, 100 nM, 200 nM, 500 nM, 1 μM to 5 μM.

Figure 4 shows the sensitivity performance of Cu^2+^ fluorescence detection on the microcapsule array chip. Six microcapsules on one chip are separately injected by six Cu^2+^ samples whose concentration range from 0 nM, 100 nM, 200 nM, 500 nM, 1 μM to 5 μM and incubated for 20 minutes. The photo in Figure 4 presents the post-injection microcapsule arrays: the fluorescence intensities of microcapsules are in a progressive tendency as Cu^2+^ concentration increases. Then software ImageJ helps calculate the fluorescence value of each microcapsule spot. And a calibration curve is fitted showing relevance between Cu^2+^ concentration and the fluorescence value. As the sensitivity test shows, quantitative detection of copper ions is feasible and the minimum detectable limit is 100 nM (100 fmol/μL), far lower than the upper limit in drinking water (~20 μM).

**Figure 5:**
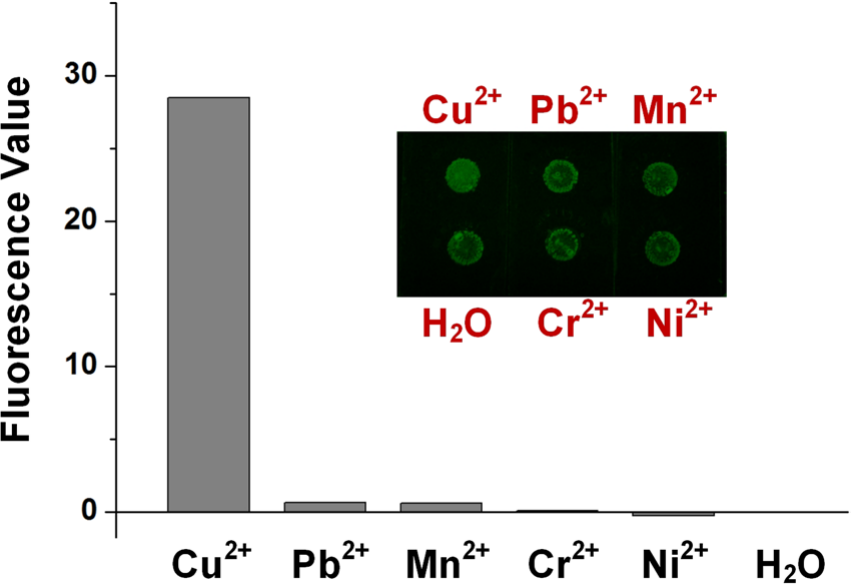
Specificity test of Cu^2+^ fluorescence detection on the microcapsule array chip. In contrast to the Cu^2+^ concentration of 5 μM, the concentrations of Pb^2+^, Mn^2+^, Cr^2+^, and Ni^2+^ are 10 μM. Inset: Photo of the specificity test.

What’s more, Figure 5 shows good specificity of our on-chip fluorescence detection, as it only targets Cu^2+^ ions rather than other mental ions such as Pb^2+^, Mn^2+^, Cr^2+^, and Ni^2+^. In contrast to the Cu^2+^ concentration of 5 μM, the concentrations of Pb^2+^, Mn^2+^, Cr^2+^, and Ni^2+^ are 10 μM. The result verifies that our detection only reacts with Cu^2+^despite the existence of other metal ions.

**Figure 6:**
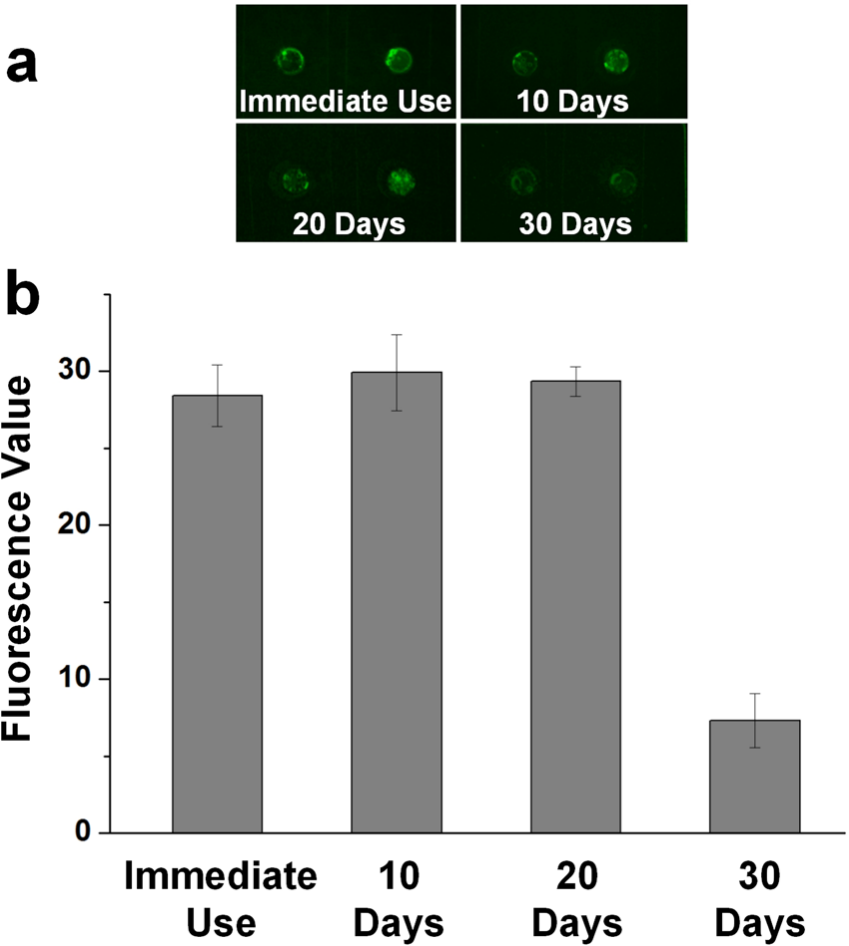
Stability test of the microcapsule array chip. The tested chips are immediately used or used after storages of 10 days, 20 days and 30 days. (**a**) Photos of the stability test. (**b**) The bar chat of measured fluorescence value, which shows the feasibility of 20-day chip storage.

As for the stability test of microcapsule arrays, the chips are stored at −20 °C for 10 days, 20days and 30 days. The test performance is shown in Figure 6. The microcapsules on the right of each photo are injected 5 μM of Cu^2+^ sample solution, whose colors turn into green after 20 minutes (a). The fluorescence value is measured and illustrated in bar chart (b), indicating that the chip is able to stay fully functional after storage of 20 days at least. This stability feature promises the microcapsule array chip a promising application prospect.

## Discussion

We successfully developed an easy-to-carry, low-cost and disposable microcapsule array chip, which can achieve ultrasensitive fluorescence quantification of copper ions. Utilizing the sensing strategy of DNAzyme, the detectable limit can be minimized to 100 nM, far lower than the upper limit of copper ions in drinking water issued by National Primary Drinking Water Regulations of USA. Compared with conventional ion determination methods, such as atomic absorption spectroscopy, our chip doesn’t require large equipment and complicated operation procedures. In addition, the 3-step usage, including syringe sample injection, transparent type sealing and transilluminator observation, is user-friendly, and the response of testing results is rapid (20 minutes), leading to the on-site detection in real time. Ice printing technique, integrated with the PS film dropping process, not only pre-seals the reaction solution, but also provides a protection of the bio-active DNAzyme system, which ensures liquid-phase detection, free contamination, and low background signals. Since ice printing is a “bottom-up” process, the fabrication is cost-effective and timesaving.

Furthermore, with fluorescence detection, other fragile system reactions and assays can take advantage of this microcapsule array chip. Combining with different fluorescence sensing strategies, multi-target detection on one chip in different microcapsules can be realized. As the photos are taken by cell phone, a cell phone application can be developed to process the results instead of ImageJ. The integration of result acquisition and processing on cell phone can make the chip usage more convenient. Thus, the multi-target detection on microcapsule arrays and cell phone integration are the main focuses of our future work.

## Methods

### Chemicals and equipment

Two DNA strands of Cu-substrate and Cu-enzyme are both synthesized by Sangon Biotech. Co., Ltd. (Shanghai, China). Cu-substrate has the sequence of 5′-AGCTTCTTTCTAATACGGCTTACC-3′, and is labeled with a FAM fluorophore (6-carboxyfluorescein) at the 3′ end and a quencher (BHQ-2) at the 5′ end. Cu-enzyme has the sequence of 5′-GGTAAGCCTGGGCCTCTTTCTTTTTAAGAAAGAAC-3′, and is labeled a 5′-quencher (Dabcyl). The 2 × DNAzyme buffer contains 200 mM HEPES, 200 mM NaCL and 200 mM KCL. HEPES is purchased from Sigma-Aldrich (St. Louis, MO, USA), and NaCL and KCL are both obtained from Sinopharm Chemical Reagent Co. Ltd (Beijing, China). Sinopharm Chemical Reagent also provides Ascorbate and CuSO4. Ultrapure water, applied for solution preparation, is purified by Milli-Q purification system (Branstead, MA, U.S.A).

Other Equipment and materials we used are listed below: microliter syringe (Anting Scientific Instrument, Shanghai, China), TJ-2A syringe pump system (Longer, Baoding, China), H2O3-PROIII dry bath (Coyote Bioscience, Beijing, China), 501S portable LED transilluminator (Landun, Beijing, China), 7 W 420 nm LED light source, three dimensional platform, Peltier cooler, silicon grease, Loctite 3311 light cure medical adhesive (Photopolymer, 3M, MN, U.S.A), Scotch 600 transparent tape (3M, MN, U.S.A), spray adhesive (3M, MN, U.S.A), poly(methyl methacrylate) (PMMA), polyethylene terephthalate (PET) film, polystyrene (PS) particles, chloroform (CHCl3). All the reagent materials are at least analytical pure.

### DNAzyme reaction solution preparation

Cu-substrate and Cu-enzyme are dissolved separately in pure water both into 10 μM. Ascorbate is dissolved in pure water into 1 mM. HEPES is diluted into 200 mM with pure water, and NaCL and KCL are dissolved both into 200 mM to prepare 2 × DNAzyme buffer. To obtain 50 μL reaction solution, 2.5 μL Cu-substrate solution, 5 μL Cu-enzyme solution, 25 μL buffer, 15 μL pure water and 2.5 μL ascorbate solution are added and mixed together. While, before the ascorbate addition, an annealing process including three stages (80 °C for 10 minutes, 65 °C for 15 minutes and 45 °C for 15 minutes) is carried out on dry bath to assist the enzyme and the substrate binding together via Watson-Crick base pairs. After the ascorbate is added, the prepared solution is ready to be used to fabricate the microcapsule arrays. The final concentrations of all reagents are listed as follows: DNAzyme buffer 1 × (contains 100 mM HEPES, 100 mM NaCL and 100 mM KCL), c (Cu-substrate) = 500 nM, c (Cu-enzyme) = 1 μM, c (Ascorbate) = 50 μM.

### Preparation of chip fabrication based on ice printing

The fabrication of the microcapsule arrays is based on our homemade ice printing system, which consists of three parts, the solution printing module, the cooling module and the environment isolating glovebox. The syringe pump system controls the volume and speed of solution printing, which guarantees a uniform shape of each printed reaction solution ice droplet. The three dimensional (X-Y-Z-axis) platform determines the location of each ice droplet. The PS solution dropping process is determined by another nozzle. The main unit of cooling module is the Peltier cooler locating on the three dimensional platform. With the heat dissipation of circulating cooling water, the chip substrate can be cooled below −20 °C. Printing and cooling modules are both placed inside a glovebox cut off from open atmosphere. Pure nitrogen is flowed into the glovebox constantly, creating a dry fabrication environment. The relative humidity inside the glovebox is as low as 0.1%, avoiding the precision interference from on-substrate frosting.

As a preparation of PS film dropping process, 27.5% PS solution is prepared by dissolving 0.55 g Polystyrene particles in 2 mL chloroform on condition that both vibration and ultrasonic condition present. Polyethylene terephthalate (PET) film with 100 μm thickness is chosen as the chip substrate, because PET is so stable that it wouldn’t be dissolved by PS solution and 100 μm is thin enough for syringe puncture. PMMA cofferdam with a window is stuck onto the PET substrate simply by spray adhesive, obtaining the basic frame of the chip. After the cleaning of PET substrate inside the cofferdam window with ethanol and pure water, the frame chip is transferred onto the Peltier cooler and is ready for ice printing fabrication. Silicon grease acts as a contact medium between the substrate and the Peltier cooler, increasing the heat conduction efficiency.

### Fluorescence determination of copper ions

The microcapsule arrays melt into liquid phase under room temperature before use. Paper tissue is used to dry the chip surface by wiping up the dew. Microliter syringe is applied to puncture the chip PET substrate and inject 1 μL target solution into each microcapsule. After cleaning the little leakage of reaction solution by paper tissue, transparent tape is used to seal the injection hole. Copper ion solutions with concentrations including 0 nM, 100 nM, 200 nM, 500 nM, 1 μM and 5 μM and 5 μM solutions of other metal ions (Pb^2+^, Mn^2+^, Cr^2+^ and Ni^2+^) are prepared in advance for quantification and specificity tests. Commercial LED transilluminator is applied to obtain the fluorescence results, which are then recorded by cell phone camera for the future advantage of on-site result processing. In this assay, photos taken by cell phone are analyzed based on the commercial software ImageJ. Utilizing ImageJ, the original RGB photo can be split into three color channels of green, blue and red, and the mean grey values of each microcapsule in green channel photo are measured representing the fluorescence intensity. Then, the fluorescence intensity of control group is normalized and a normalization factor is obtained. Based on this factor, the normalized fluorescence intensity of experimental group can be calculated, and the fluorescence value of experimental group is equal to its normalized fluorescence intensity minus that of control group. Copper ions in the sample can be quantified on account of the processed fluorescence value, since it indicates the intensity of fluorescence signal enhanced by the copper ion injection. This measuring process is illustrated in Figure S2. Instead of ImageJ, the development of a cell phone application integrating both acquisition and processing of the detection is a promising goal in the future.

## Electronic supplementary material

Supplementary information

## Data Availability

The authors have the copyrights of all materials and data in this article, including the figures.
