# Supplementary material for: Rapid Quantitative Fluorescence Detection of Copper Ions with Disposable Microcapsule Arrays Utilizing Functional Nucleic Acid Strategy
Source: Sci Rep. 2019 Jan 10;9:36. doi: 10.1038/s41598-018-36842-x (PMC6328549; doi:10.1038/s41598-018-36842-x)
Supplement: Supplementary file 1 — Supplementary information [file 41598_2018_36842_MOESM1_ESM.docx]

Rapid Quantitative Fluorescence Detection of Copper Ions with Disposable Microcapsule Arrays Utilizing Functional Nucleic Acid Strategy

**Enqi He ^1,4^, Liangyuan Cai ^2^, Fengyi Zheng^3^, Qianyu Zhou ^2^, Dan Guo^1^, Yinglin Zhou^2,*^, Xinxiang Zhang^2,*^, and Zhihong Li^3,4,*^**

^1^State Key Laboratory of Tribology, Department of Mechanical Engineering, Tsinghua University, Beijing, 100084, China

^2^Beijing National Laboratory for Molecular Sciences (BNLMS), Key Laboratory of Bioorganic Chemistry and Molecular Engineering of Ministry of Education, College of Chemistry, Peking University, Beijing, 100871, China

^3^National Key Laboratory of Science and Technology on Micro/Nano Fabrication, Institute of Microelectronics, Peking University, Beijing, 100871, China

^4^Center for Nano and Micro Mechanics, Tsinghua University, Beijing, 100084, China

* [zhhli@pku.edu.cn](mailto:zhhli@pku.edu.cn); [zhouyl@pku.edu.cn](mailto:zhouyl@pku.edu.cn); [zxx@pku.edu.cn](mailto:zxx@pku.edu.cn)

**Table of Contents**

- **Supplementary Figure S1-S3**


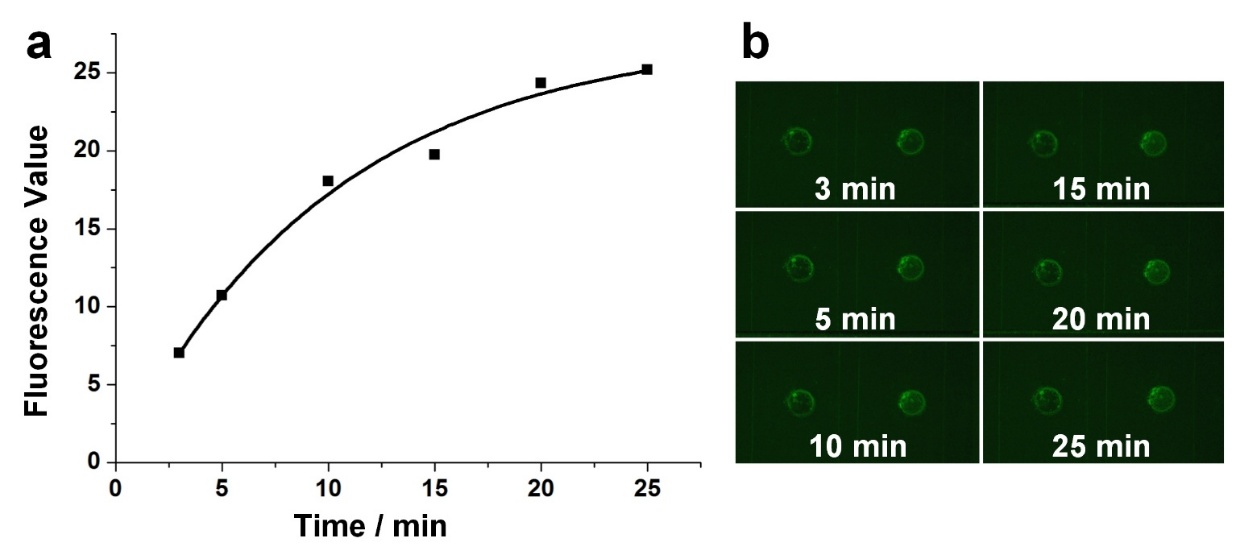


Figure S1. The variation test of fluorescence signal against reaction time. The concentration of injected copper ion is 5 μM and the fluorescence signal results are detected and measured 3 minutes, 5 minutes, 10 minutes, 15 minutes, 20 minutes and 25 minutes after sample injection. (a) The calibration curve. (b) The real photos.


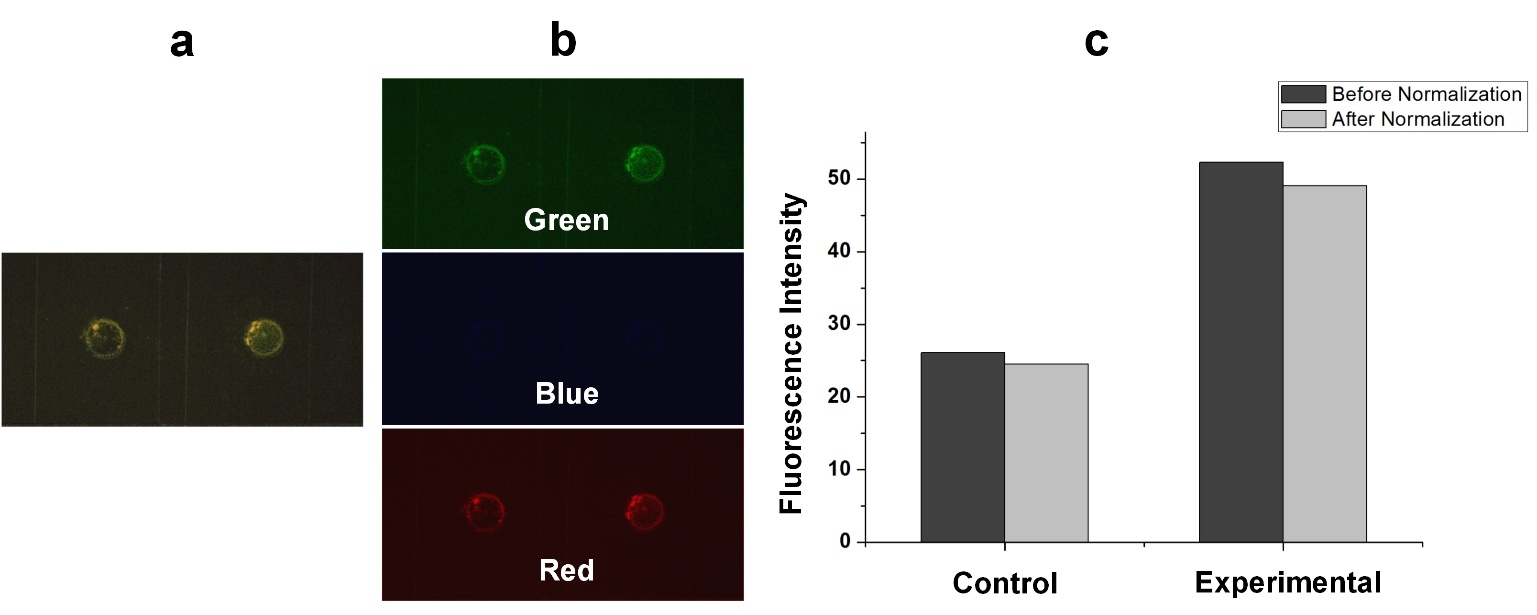


Figure S2. The measurement of fluorescence value, which indicates the intensity of fluorescence signal enhanced by the copper ion injection. Utilizing ImageJ, the original RGB photo can be split into three color channels of green, blue and red, and the mean grey values of each microcapsule in green channel photo are measured representing the fluorescence intensity. Then, the fluorescence intensity of control group (microcapsule injected with pure water) is normalized into a constant number of 24.5 obtaining a normalization factor. Based on this factor, the normalized fluorescence intensity of experimental group can be calculated. The fluorescence value of experimental group is its fluorescence intensity minus that of control group. (a) The original RGB photo of microcapsules injected with pure water (left) and 5 μM copper ion sample (right). (b) The split photos of Fig. S2(a) in green, blue and red channels. (c) The bar chart of fluorescence intensity measured and calculated based on the green channel photo before and after normalization.


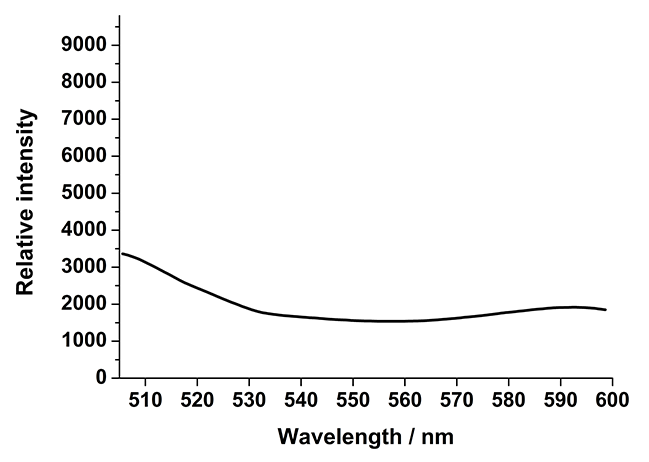


Figure S3. Fluorescence emission spectrometry of the solidified photopolymer. Excitation wavelength is 480 nm.
